# High prevalence of atrial fibrillation among Greenlanders with ischemic stroke – atrial fibrillation found in more than 30% of cases

**DOI:** 10.3402/ijch.v72i0.22628

**Published:** 2013-11-22

**Authors:** Karen Bjorn-Mortensen, Folmer Lynggaard, Michael Lynge Pedersen

**Affiliations:** 1 Queen Ingrid's Health Center, Nuuk, Greenland; 2 Greenland Center for Health Research, University of Nuuk, Nuuk, Greenland; 3 Department of Internal Medicine, Queen Ingrid's Hospital, Nuuk, Greenland

**Keywords:** atrial fibrillation, ischemic stroke, risk factor

## Abstract

**Objectives:**

To estimate the prevalence of atrial fibrillation among Greenlanders with ischemic stroke.

**Study design:**

A cross-sectional study.

**Methods:**

Information on atrial fibrillation and vitamin K antagonistic treatment at admittance and at discharge was obtained for Greenlanders admitted to Queen Ingrid's Hospital in Nuuk with an ischemic stroke in 2011 or in 2012 with methods described in details elsewhere.

**Results:**

Of 139 patients (64 males and 75 females) Greenlanders with an ischemic stroke in 2011 (n=74) or 2012 (n=65), 5.0% (n=7) had known atrial fibrillation prior to stroke compared to 32.4% (n=45) after discharge (p<0.01).

**Conclusions:**

More than 30% of ischemic stroke patients in this study had atrial fibrillation and only 5% were diagnosed prior to the stroke, suggesting that unknown atrial fibrillation is a substantial risk factor of ischemic stroke among Greenlanders.

Ischemic stroke has been reported as common among Greenlanders (1). Furthermore, a recent study has reported stroke incidence to be high among younger Greenlanders (2). Risk factors of ischemic stroke are similar to those of cardiovascular disease. Non-modifiable risk factors include age, gender, and genetics and modifiable risk includes smoking, hypertension, obesity, diabetes, physical inactivity and hyperlipidaemia. In addition, earlier stroke or transient ischemic attack, carotic stenosis, heart disease and atrial fibrillation are considered risk factors.

However, the prevalence of cardiovascular risk factors among Greenlanders with ischemic stroke is unknown. The aim of this study was to estimate the prevalence of atrial fibrillation among Greenlanders with ischemic stroke.

## Methods

This study was carried out as a cross-sectional study, including all Greenlanders admitted to Queen Ingrid's Hospital in Nuuk with an ischemic stroke in 2011 or in 2012. Greenlanders were defined as persons born in Greenland. Information about atrial fibrillation and vitamin K antagonistic treatment at admittance and at discharge from the hospital was obtained from patient hospital files, discharge letters, the electronic laboratory system and the electronic medical record used in the primary care system. Methods are described in details elsewhere (2).

## Results

A total of 139 patients (64 males and 75 females) Greenlanders with a median age of 62 years at time of diagnosis (interquartile range: 54–71 years) were identified with an ischemic stroke in 2011 (n=74) or 2012 (n=65). This corresponds to an incidence of 138 patients/year/100,000 inhabitants (95% CI 115–161).

As shown in Table I, the prevalence of known atrial fibrillation was 5.0% (n=7) prior to stroke compared to 32.4% (n=45) after discharge from QIH (p<0.01). Correspondingly, the number of patients receiving vitamin K antagonistic treatment increased from 2.9 to 27.3% during hospitalization.

**Table I:** Atrial fibrillation and vitamin K antagonistic treatment prior to stroke and after hospitalization among Greenlandic stroke patients in Greenland 2011–2012

	Prior to stroke n (%)	After hospitalization n (%)	p
Study population	139	139	
Atrial fibrillation	7 (5.0)	45 (32.4)	<0.01
Vitamin K antagonists	4 (2.9)	38 (27.3)	<0.01

## Discussion

The prevalence of atrial fibrillation among Greenlanders with ischemic stroke was high, since more than 30% of the patients were diagnosed with atrial fibrillation during hospitalization – mostly undiagnosed prior to stroke.

A previous study among ischemic stroke patients in Greenland reported smoking as an associated risk factor (1). A large worldwide study concluded that 5 risk factors (hypertension, current smoking, abdominal obesity, diet, physical activity) accounted for more than 80% of all risk. Among ischemic stroke patients, 14% had a cardiac cause including atrial fibrillation (3). Since more than 30% of ischemic stroke patients in this study had atrial fibrillation and only 5% were diagnosed prior to the stroke, the study suggests that undiagnosed atrial fibrillation is a substantial risk factor of ischemic stroke among Greenlanders. The frequent atrial fibrillation makes early diagnosis and treatment of atrial fibrillation important in the prevention of stroke in Greenland.
